# Integrated phytoremediation focused on microbial investigation

**DOI:** 10.1039/c7ra05895k

**Published:** 2018-01-25

**Authors:** T. Y. Yeh, C. M. Kao, W. H. Chen

**Affiliations:** Department of Civil and Environmental Engineering, National University of Kaohsiung Kaohsiung 811 Taiwan tyyeh@nuk.edu.tw +886-7-591-9376 +886-7-591-9536; Institute of Environmental Engineering, National Sun Yat-Sen University Kaohsiung Taiwan

## Abstract

Phytoremediation is an environmentally friendly green rehabilitation technology that is often incorporated with an application to improve phytohormones required for the growth of agricultural plants with the expectation to improve the effectiveness of plant rehabilitation. This study adopts phytoremediation, a green remediation technology, for the sake of restoring soil fertility and ensuring environmental sustainability, and adds ethylenediaminedisuccinic acid (EDDS) and the plant growth regulator (GA_3_) to examine the overall efficiency of phytoremediation. The experiments using pots in this study finds that environmentally sustainable phytoremediation achieves the greatest efficacy regarding the remediation of soil polluted by copper, zinc and nickel. The best combination of operational factors is the addition of the EDDS and GA_3_. The environment where the EDDS is added shows a poorer performance in the remediation of the heavy metal lead. In addition, the PCR(Polymerase chain reaction)-DGGE analysis results of bacterial flora change show that the combination “heavy metal + EDDS + GA_3_” brings about the richest bacterial flora, indicating that the addition of EDDS and GA_3_ can stimulate microbial growth, thereby achieving richer bacterial flora.

## Abstract 2

Phytoremediation is a green rehabilitation technology that is often incorporated with an application to improve phytohormones required for the growth of agricultural plants with the expectation to improve the effectiveness of plant rehabilitation.

## Graphical abstract

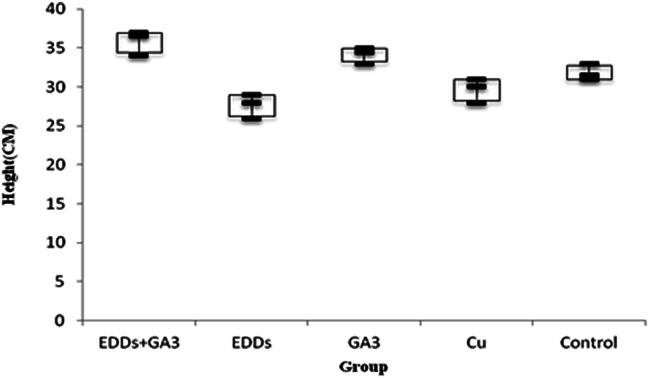

## 1. Introduction

In Taiwan, the main technologies currently adopted regarding the remediation of soil polluted by heavy metals include blending and dilution, soil acid washing, as well as soil removal and replacement. The blending and dilution method is often used for farmland with deeper soil and a lower concentration of pollutants. The standard procedure for such remediation is easy to establish and implement, and therefore more favorable in terms of cost and time control. Nevertheless, during the process, not all parts of the soil can be fully mixed, which causes the failure of soil improvement. Soil acid washing is generally applied to farmland with high pollutant concentrations by using nitric acid, hydrochloric acid, and citric acid for oxidation reduction and complexation processes in the farmland. It can reduce concentration levels of heavy metals in the soil in a short period of time, and the acid solution can be recycled to lower the overall costs after the remediation. However, such a method could alter soil pH value, which leads to a certain level of damage to farmland and infertility. Soil removal and replacement excavates soil with high concentrations of heavy metal pollutants, and replaces it with clean soil. But such a method is more expensive in a sense that clean soil needs to be purchased, and waste soil needs to be purified.

Compared with the above-mentioned methods, phytoremediation has a wider application for the remediation of polluted soil and underground water. It is an eco-friendly green remediation technique focusing on the sustainability of environmental and ecological resources. Since plants for phytoremediation can be used to absorb and store heavy metals from the soil at polluted sites, it is also more likely to be accepted by the general public than conventional remediation. Additionally, phytoremediation is more cost-effective and suitable for complex compositions of both organic and inorganic metal pollutants in sediment or soil. Furthermore, it will not damage soil structure and texture, and people living in the remediation area will demonstrate higher acceptance toward this method. It also improves the scenery, provides more added value and function, and creates a sustainable environment.^1^

Consequently, sunflowers are selected for phytoremediation in this study. By integrating a plant growth hormone, GA_3_, as well as environmentally friendly and bio-degradable chelating agent, EDDS, this study aims to improve the effectiveness of the heavy metal contamination remediation *via* phytoremediation, thereby achieving environmental sustainability.

## 2. Materials and method

### 2.1 Pot experiment

The operational conditions are shown in Table 1. First, the soil from the campus of National University of Kaohsiung and non-organic soil purchased from a gardening store are adopted in the experiment. Table 2 shows the soil parameters. Before the experiment, each pot, with the dimensions of 70 cm long × 30 cm wide, was filled with 10 kg soil. After filling the pots, heavy metals of Cu, Zn, Pb, and Ni were added with a concentration level one or two times higher than the regulatory standards announced by the Environmental Protection Administration. Concentration levels were kept low enough to prevent the sunflower from dying. The concentration levels of added heavy metals were Cu (800 mg kg^−1^), Zn (4000 mg kg^−1^), Pb (4000 mg kg^−1^), and Ni (4000 mg kg^−1^), respectively. After the heavy metal solutions were added, we air dried the experimental soil and added 100 ml EDDS with a concentration level of 500 μmol kg^−1^, then had it air dry again. Afterwards, we transplanted four sunflowers to each experiment pot. The sunflowers were exposed to sunshine in a cycle of 16 h/8 h (day/night). During the experiment, 50 ml GA_3_ and 500 ml water were sprayed once every morning and evening, and the growth of the sunflowers was measured and recorded with photos every half month (15 days). The experiment lasted one month (30 days).

**Table 1:** Operational condition of pot experiment

Parameters	Experimental condition
Plants in pot	Sunflower (helianthus annuus)
Concentration of heavy metals	Cu (800 mg kg^−1^), Zn (4000 mg kg^−1^), Pb(4000 mg kg^−1^), Ni (400 mg kg^−1^)
GA_3_ concentration	10^−8^ mol kg^−1^
EDDS concentration	500 μmol kg^−1^
Sunshine	16 h/8 h day/night cycle
Experiment duration	30 days
Pot dimension	70 cm long × 30 cm wide

**Table 2:** Background parameters of experimental soil

Soil parameters	Low organic matter soil
pH	6.73 ± 0.16
Organic substance (%)	4.16 ± 0.27%
Background concentration of heavy metal, Cu, in soil (mg kg^−1^)	87.1 ± 5.6
Background concentration of heavy metal, Zn, in soil (mg kg^−1^)	133.5 ± 7.3
Background concentration of heavy metal, Pb, in soil (mg kg^−1^)	0 ± 0.01
Background concentration of heavy metal, Ni, in soil (mg kg^−1^)	9.2 ± 1.1

### 2.2 Analysis of heavy metals

Pot sunflowers were separated into roots, stems, leaves, and petals, and then baked in an oven at 104 °C for 24 hours before being cooled at room temperature. After being ground, 5.5 ml nitric acid and 0.5 ml hydrochloric acid respectively were added to 0.5 g plant parts for extraction. They were then microwave-digested using a MarsX microwave digester before being analyzed with the AA.

### 2.3 Assessment of phytoextraction efficiency

Bioconcentration factor indicates the efficiency of a plant species in accumulating a metal into its tissues from the surrounding environment.^2^ It is calculated as follows.^3^
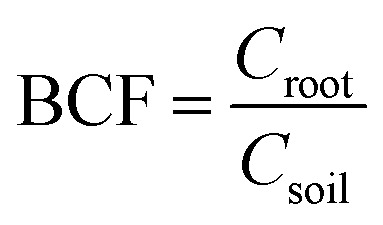


Translocation factor indicates the efficiency of the plant in translocating the accumulated metal from its roots to shoots. It is calculated as follows.^4^
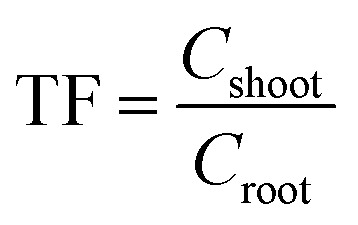


Phytoremediation efficiency factor was calculated for pot experiment results to illustrate the phytoextraction efficiency.
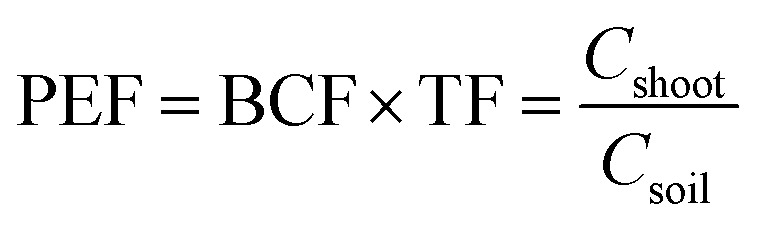
In the above formula, *C*_soil_, *C*_root_ and *C*_shoot_ represent the concentration of the soil, underground part of plants (*i.e.* the roots) and above parts of plants (*i.e.* the shoots and leaves), respectively.

### 2.4 Bacterial flora analysis

#### 2.4.1 Denaturing gradient gel electrophoresis (DGGE)

The soil sampling for the pot experiment, which contained Cu, Zn, Pb, and Ni with concentration levels two times higher than the regulatory standards, was conducted by harvesting the sunflowers and collecting 100 g of soil respectively from each pot. After mixing soil to make it even, 100 g soil was collected for bacterial flora analysis. During the undertaking of the experiment, glass plates were disinfected with alcohol, and placed on the holders of the gel caster covered with sponge before being stabilized with clamps. Afterward, the bottom gel was produced. First, we prepared 0.1 g 10% APS (ammonium persulfate) with 1 ml of deionized water, and added reagents to a centrifuge tube in the following order: 1.68 g Urea, 1.6 ml foramide, 1.2 ml acrylamide/bis, and 0.1 ml 50× TAE buffer; we then added ddH_2_O to a total volume of 16 ml, and finally added 5.3 μl TEMED and 53 μl of 10% APS. After mixing well, we poured 1,600 μl of the solution onto the gel caster and further pressed 1,600 μl of isopropanol onto the top of the gel. We then waited about 30 minutes. After the bottom gel was desiccated, we decanted the isopropanol. Then the two types of top gel with different concentration levels were produced respectively in the following steps. First of all, for the top gel with a 35% concentration, we added 2.25 g urea, 2.40 ml foramide, 2.4 ml acrylamide/bis, and 0.32 ml 50× TAE buffer. Then, we added ddH_2_O to a total volume of 16 ml, followed by the addition of 6.4 μl TEMED and 64 μl 10% APS. For the top gel with a 57.5% concentration, we added 3.86 g urea, 3.68 ml foramide, 2.4 ml acrylamide/bis, and a 0.32 ml 50× TAE buffer. Then, ddH_2_O was added to a total volume of 16 ml, with 6.4 μl TEMED and 64 μl 10% APS added finally. After preparation, the solutions were properly mixed and kept in ice at a low temperature. Gels with two different concentration levels were prepared and put into syringes, which were then placed on the platform for gradient formation. After setting them aside for about 15 minutes, we inserted combs and added a small amount of APS to each well to facilitate polymerization. After about two hours, when the gel had polymerized, we pulled out and cleaned the combs with ddH_2_O, and used syringes to draw out the liquid inside the wells. Then, we placed the gel onto the platform before adding PCR products to the wells. Polymerase chain reaction (PCR) is a technique used in molecular biology to amplify a single copy or a few copies of a piece of DNA across several orders of magnitude, generating thousands to millions of copies of a particular DNA sequence.

Purified PCR products were quantified and sized. After that, they were added to the denaturing gradient gel for DGGE preparation. First, we turned on the heating system of the electrophoresis tank to preheat the running buffer (1× TAE buffer) to 60 °C so that the DNA could undergo the denaturing gradient effect at a stable temperature during the process. Then Perist, a liquid cycling system, was turned on and the denaturing gradient gels were put into the electrophoresis tank. Afterwards, the PCR products mixed with loading dye were put in the groove above the denaturing gradient gel. Finally, electrophoresis was conducted with a lower voltage of 65 volts at a temperature of 60 °C for 14.5 hours.

#### 2.4.2 SYBR green I for DNA staining

In order to have the most complete examination on the bacterial flora of microorganism in soil, we increased not only the DNA contents but also chose a DNA staining dye with higher sensitivity. Normally, DNA staining dyes, in the order of sensitivity, are SYBR green I > silver staining > ethidium bromide. Thus, this experiment adopted SYBR green I for DNA staining.^5^ The denaturing gradient gels were carefully extracted from the electrophoresis system and placed on trays. They were then cleaned with deionized water more than three times. Afterward, SYBR green I staining was conducted; we put the denaturing gradient gels in the 200 ml 1× TAE buffer solution with 20 μl SYBR green I (amresco), and shook the solution at the speed of 150 rpm for 1 hour. When SYBR green I staining is being conducted, treatment should occur away from light. After staining, the LAS-3000 Luminescent image analyzer (FUJIFILM) was used for fluorescence excitation and image capture.

## 3. Result and discussion

### 3.1 Correlation analysis of plant growth differences and concentration levels of heavy metals in soil

**Fig. 1:**
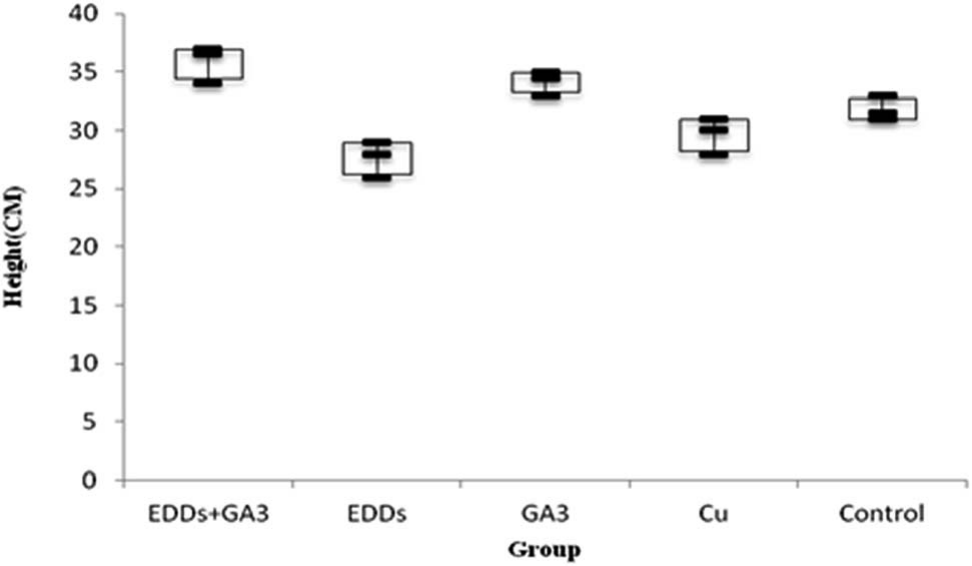
Comparison of total plant growth in height among the groups with Cu added.

As shown in the comparison of each group's overall growth in height (Fig. 1), the group with GA_3_ added showed greater height and weight growth in comparison to the group with only Cu added, which proved the effectiveness of adding GA_3_ to promote plant growth; meanwhile, the group with EDDS added showed less growth in height and weight in comparison to the others. Previous research indicated that a chelating agent can assist in increasing the absorption of heavy metals in plants. However, it can also increase toxicity in plants.^6^ Thus, the group with EDDS added showed less growth in height and weight in comparison to the others. However, the group with EDDS + GA_3_ added grew taller and heavier than the group with EDDS added, which indicated that the addition of GA_3_ can effectively protect sunflowers from the impact of adding EDDS.

Hence, according to the comparison of total growth in height among the groups with Cu added (Fig. 1), the group with EDDS + GA_3_ is more suitable for application to the phytoremediation of soil at a concentration level of Cu.

**Fig. 2:**
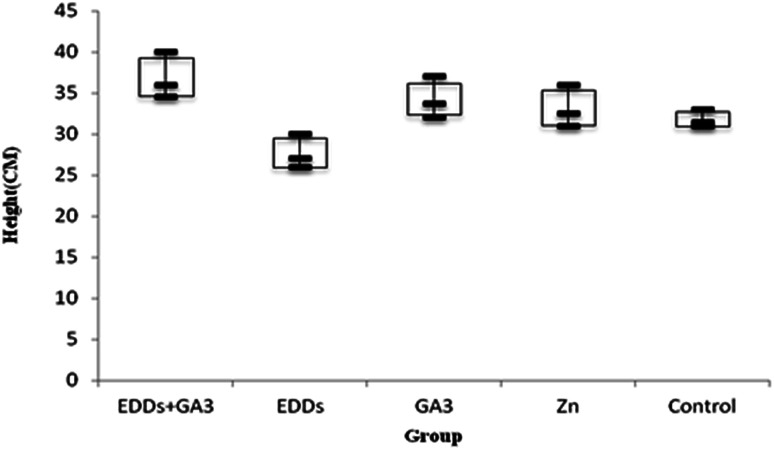
Comparison of total plant growth in height among the groups with Zn added.

As shown in total growth of height comparisons for each group (Fig. 2), the group with EDDS added showed inhibited plant growth in the soil with a concentration level of Zn. Among the groups in the experiment, the group with EDDS + GA_3_ added showed greater growth in weight and height than that of the other groups. EDDS in the soil with Zn added at a concentration still promotes the growth of plant to a certain level, which indicates that Zn is not so toxic to sunflowers, and that the addition of GA_3_ to the soil added with Zn at a concentration can effectively inhibit the disadvantage of a chelating agent to plant growth.

Hence, after comparing height and weight for plant growth, the group with EDDS + GA_3_ added is more suitable for application to the phytoremediation of soil at a concentration level of Zn.

**Fig. 3:**
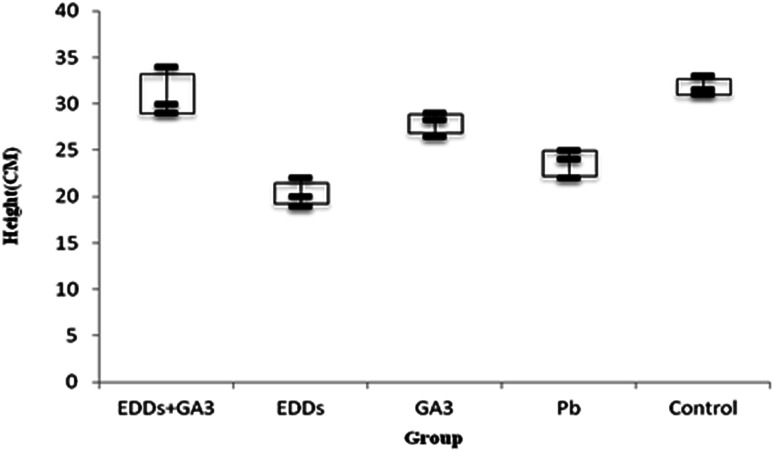
Comparison of total plant growth in height among the groups with Pb added.

As shown in the comparison of total growth in height among each group (Fig. 3), the group with EDDS showed significantly lower growth in plant height in the soil with concentration level of Pb than the group with GA_3_ and the group with EDDS + GA_3_. The result indicates that GA_3_ can still effectively inhibit the disadvantage of a chelating agent for plants grown in the soil with Pb at a concentration. Besides, the group with EEDS showed lower growth in plant height than the group with only Pb, which explains why the addition of a chelating agent can increase the effectiveness of sunflowers in absorbing heavy metals, but also increase the toxicity to sunflowers.

Hence, after comparing plant height and weight growth, the group with EDDS + GA_3_ is more suitable for application to the phytoremediation of soil at a concentration level of Pb.

**Fig. 4:**
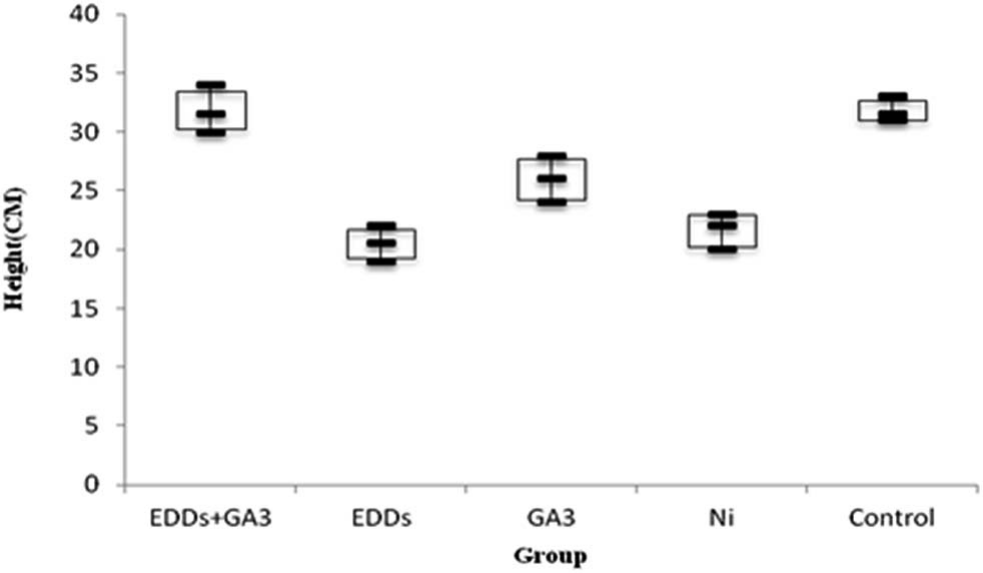
Comparison of total plant growth in height among the groups with Ni added.

As shown in totals for comparisons of how much each group grew (Fig. 4), the group with EDDS inhibits plant height growth in the soil with a concentration level of Ni. Among the groups in the experiment, the group with GA_3_ and the group with EDDS + GA_3_ grew significantly higher than the group with EDDS and the group only with Ni.

Hence, according to the comparison of total height among the groups with Ni added (Fig. 4), the group with EDDS + GA_3_ is more suitable for application to the phytoremediation of soil at a concentration level of Ni one time higher than regulatory standards.

### 3.2 Discussion on the concentration of soil and the absorbed heavy mental of sunflowers

We examined the total heavy metal contents of sunflower plants. From the comparison of total heavy metals absorbed by plants in each group (Table 3), we were able to determine total heavy metals absorbed by sunflowers planted in soil containing Cu, Zn, Pb, and Ni at a concentration level two times higher than regulatory standards during the phytoremediation in this experiment. First, we compared the groups using EDDS + GA_3_ to the groups with only heavy metals, and we found that, in the soil containing Cu, Zn, Pb, and Ni at the concentration level two times higher than regulatory standards, the former indeed saw an increase in overall heavy metal accumulation in the sunflower, especially for the soil containing Cu, Zn, and Ni. However, there was no significant difference for the soil containing Pb.

**Table 3:** Pot experiment: total heavy metal contents absorbed by sunflowers in each group

Group	EDDS + GA_3_	EDDS	GA_3_	Heavy metal only
Cu	172.4 ± 3.9 mg kg^−1^	121.9 ± 4.7 mg kg^−1^	90.2 ± 5.6 mg kg^−1^	80.9 ± 4.0 mg kg^−1^
Zn	1005.6 ± 21.3 mg kg^−1^	547.4 ± 13.2 mg kg^−1^	411.2 ± 11.2 mg kg^−1^	325.9 ± 7.2 mg kg^−1^
Pb	88.6 ± 5.4 mg kg^−1^	63.3 ± 3.1 mg kg^−1^	49.1 ± 4.7 mg kg^−1^	46.2 ± 2.3 mg kg^−1^
Ni	161.2 ± 5.9 mg kg^−1^	137.0 ± 3.6 mg kg^−1^	143.2 ± 4.1 mg kg^−1^	71.4 ± 3.5 mg kg^−1^

Next is the comparison between the use of EDDS + GA_3_ and the addition of GA_3_ only. The comparison of heavy metal absorption in each group of sunflowers (Table 3) indicates the group with GA_3_ absorbs lower total volumes of heavy metals than the groups with EDDS + GA_3_ and with EDDS only. Since EDDS can improve the mobility of heavy metals in soil and the heavy metal absorption of roots, the total volume of heavy metal absorption for the group with GA_3_ is lower than those with EDDS + GA_3_ and with EDDS. The difference is especially evident in the soil containing Cu, Zn, and Ni, but the difference in the soil containing Pb is not obvious.

### 3.3 Discussion on phytoremediation efficacy in soil at a concentration

First, the BCF values shown in Table 4 mainly present the comparison of heavy metal accumulations in roots, as well as the heavy metal content in soil. Higher BCF values indicates better heavy metal absorption. We compared the BCF values of groups with only heavy metals to those with EDDS, with GA_3_ and with EDDS + GA_3_. Table 4 present the comparison of the soil for concentration levels of Cu, Zn, and Pb. The result shows that the BCF value of the EDDS + GA_3_ group is the highest, and there is no significant difference for the groups with EDDS + GA_3_ at concentration levels of Cu, Zn, and Pb. Such results indicate the addition of EDDS and GA_3_ together can effectively improve the heavy metal absorption of sunflower roots, and the efficacy of the roots' absorption remains unchanged as the concentration of heavy metals increases. After comparing Table 4, we found that the group with EDDS show optimal performance, followed by those with EDDS + GA_3_ in the soil at a concentration level of Ni. The results show the addition of EDDS in the soil containing Ni actually improves sunflower root absorption.

**Table 4:** Pot experiment: BCF value comparison at a concentration

Group	EDDS + GA_3_	EDDS	GA_3_	Heavy metal only
Cu	0.26	0.20	0.11	0.12
Zn	0.29	0.16	0.10	0.11
Pb	0.05	0.03	0.01	0.02
Ni	0.37	0.52	0.36	0.26

TF value refers to the comparison of heavy metal contents of parts above the ground (stem, leave, and petal) with those of roots in order to examine the mobility of heavy metals from roots to parts above the ground. Higher TF value indicates better mobility from roots to parts above the ground. Since GA_3_ promotes the growth of plant parts above the ground by bringing more nutrition from roots, xylem, to the parts above the ground, we examine its efficacy to see if it brings nutrition and heavy metals together to parts above the ground. According to Table 5, the group with GA_3_ has the highest TF value in soil at the concentration level of Cu, indicating the adding GA_3_ effectively promotes the mobility of Cu to sunflower parts on the ground. However, when comparing Table 5, we found there is no significant difference among the group with EDDS + GA_3_, the group with GA_3_, and the group with heavy metals only. This also proves the absorption efficacy of sunflower parts is not reduced in the soil containing heavy metals. Furthermore, when comparing Table 5 to examine the absorption efficacy of Zn in soil at a concentration, we found the value of GA_3_ group increases greatly as the concentration level of Zn increases in the soil, indicating the addition of GA_3_ can effectively increase Zn mobility of the sunflower and the absorption of sunflower parts above the ground. Table 5 also show, in comparing the data of concentration levels of Pb, there are no significant differences for values among most of the groups, except the group with GA_3_. Finally, comparing the soil at the concentration level of Ni, the data shown in Table 6 indicates that the TF values of the most of the groups reduce when the concentration of Ni in the soil increases, but the group with EDDS rises when the concentration increases. This indicates the addition of EDDS can effectively assist sunflowers in transmitting Ni from roots to parts above the ground.

**Table 5:** Pot experiment: TF value comparison at a concentration

Group	EDDS + GA_3_	EDDS	GA_3_	Heavy metal only
Cu	2.30	2.09	3.23	2.26
Zn	2.39	2.44	3.25	2.03
Pb	0.82	0.96	1.51	1.16
Ni	3.32	1.63	2.98	1.78

**Table 6:** Pot experiment: PEF value comparison at a concentration

Group	EDDS + GA_3_	EDDS	GA_3_	Heavy metal only
Cu	0.60	0.41	0.35	0.28
Zn	0.71	0.39	0.31	0.22
Pb	0.04	0.03	0.03	0.02
Ni	1.24	0.85	1.07	0.46

The last item we would like to examine is PEF value, the comparison of overall phytoremediation. The data shown in Table 6 present the comparison of the overall efficacy of the phytoremediation at the concentration levels of Cu, Zn, Pb and Ni, and the result shows adding EDDS and GA_3_ together is optimal for phytoremediation. In addition, the data shown in Table 6 present that there was no significant difference for PEF values between the soil at the concentration level of Cu, Zn, and Pb. The result indicates that even if the concentration level of Cu, Zn, and Pb in the soil increases by two times, there are no significantly increasing or reducing efficacy for overall phytoremediation; however, when the concentration level of Ni is increased by two times, the efficacy is greatly reduced relatively. This indicates that the efficacy of the phytoremediation will decrease when the concentration of Ni increases.

From the above results we can draw conclusions concerning the optimal operating parameters for sustainable phytoremediation. In the soil containing Cu, Zn, and Ni, the addition of EDDS and GA_3_ together shows much higher PEF values than that of any of other groups. Thus, adding EDDS and GA_3_ together is a suitable option for the phytoremediation of Cu, Zn, and Ni. As to the soil containing Pb, we need to look for another means of remediation which has more efficiency.

In conclusion, the above results prove the addition of EDDS and GA_3_ for phytoremediation can be more helpful to the heavy metal absorption and accumulation of plants in comparison to conventional phytoremediation. It can also improve the efficiency of remediation, and shorten the required time for the whole procedure.

### 3.4 Discussion on impact of phytoremediation on heavy metal-polluted soil and bacterial flora

**Fig. 5:**
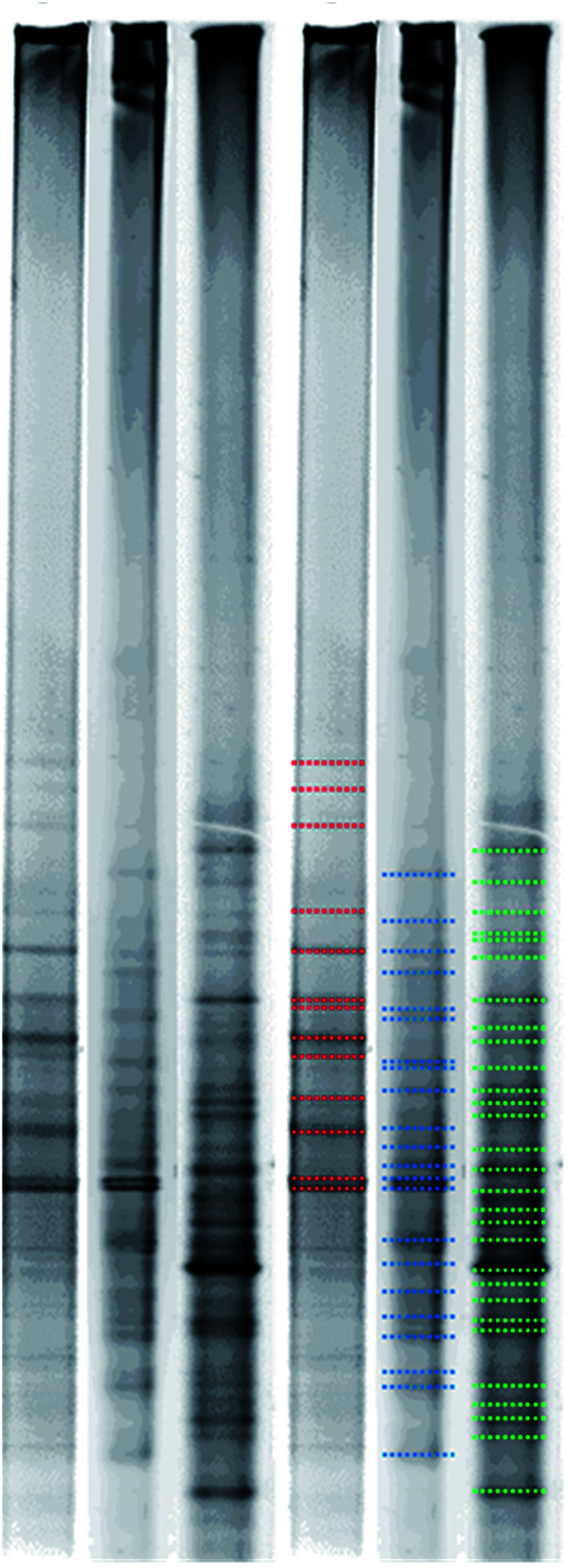
DGGE bacterial flora analysis of test groups.

Fig. 5 shows bacterial flora change in three test groups after the PCR and DGGE analysis. According to Fig. 5, there are 13 bands of the heavy metal + H_2_O (marked with red color); there are 22 bands of heavy metals + GA_3_ (marked with blue color); and there are 29 bands of heavy metals + EDDS + DGA_3_ (marked with green color). DGGE analysis is conducted by using gradient screening after enlarging and purifying DNA sections. When a band appears, it indicates the dominant bacteria group. As a result, the more bands in a sample, the richer bacterial flora in the sample, and the higher possibility of diverse microorganisms.

## 4. Conclusion and suggestion

### 4.1 Conclusion of pot experiment

The pot experiment in this study examines and determines the optimal operational group. The researchers planted sunflowers in the soil containing Cu, Zn, Pb, and Ni, and compared their growth status, total contents of heavy metals in their roots, as well as parts above the ground (stem, leave, and petal), then examined the effective coefficient of phytoremediation. The results show the addition of EDDS can effectively promote the heavy metal mobility, and increase the heavy metal absorption of plants; while the addition with GA_3_ can increase the biomass of plants and improve upward mobility of plants. Finally, the pot experiment indicates that adding EDDS and GA_3_ together can generate the best absorption efficacy in the soil containing Cu, Zn, and Ni.

As to the selection of a chelating agent, the use of EDDS has the best effect on remediation for the soil containing Cu, Zn, and Ni. Moreover, EDDS can be decomposed by microorganisms in the soil. Thus, it will not result in acid soil or secondary pollution like a chemical chelating agent. However, EDDS has less efficacy for soil polluted by Pb. As a result, we suggest looking for other biodegradable chelating agents to remedy Pb polluted soil more effectively.

Consequently, the pot experiment demonstrates the environmentally sustainable phytoremediation possesses optimal efficacy in remedying soil polluted by Cu, Zn, and Ni, and the optimal method is adding EDDS and GA_3_ together. However, EDDS is not suitable for the remediation of Pb polluted environments.

### 4.2 Conclusion of the impact of phytoremediation on bacterial flora in soil

According to bacterial flora change result acquired from PCR and DGGE analysis, among the three groups, heavy metal + H_2_O, heavy metal + GA_3_, and heavy metal + EDDS + GA_3_, the last group has the richest bacterial flora. This demonstrates that when EDDS and GA_3_ are added together, the growth of microorganisms will be stimulated to create the richer development of bacterial flora.

## Conflicts of interest

There are no conflicts to declare.

## Supplementary Material
